# Quadrant training: a simple typology-based approach to improve strength and endurance in youth soccer players

**DOI:** 10.3389/fspor.2026.1936767

**Published:** 2026-10-07

**Authors:** Timo Steidle, Benedikt Meixner, Mascha Lenk, Marcelle Schaffarczyk, Billy Sperlich

**Affiliations:** 1 Integrative and Experimental Exercise Science and Training, Department of Sport Science, University of Würzburg, Würzburg, Germany; 2 Institute of Sport Science, University of the Bundeswehr Munich, Neubiberg, Germany

**Keywords:** 30–15 intermittent fitness test, countermovement jump, group-based training approach, isometric mid-thigh pull, lactate threshold

## Abstract

**Introduction:**

Youth soccer players show heterogeneous performance profiles, yet individualized conditioning is often impractical outside professional settings. We examined whether a typology-based training allocation into four quadrants (“Quadrant Training”; QT) improves endurance- and strength-related performance compared with regular soccer training alone.

**Methods:**

Male U16-U17 players from two youth academies completed a non-randomized, longitudinal controlled intervention (academy-level and intervention effects are confounded); one academy served as intervention group (*n* = 38), the other as control (*n* = 22). Players were classified into four endurance quadrants, i.e., below and above team mean based on 30-15 Intermittent Fitness Test velocity [vIFT] and velocity at 4 mmol·L⁻¹ blood lactate [V4] and four strength quadrants based on relative isometric mid-thigh pull strength [IMTP] and countermovement jump height [CMJ], completing quadrant-specific endurance training plus one weekly strength session.

**Results:**

Between-group ANCOVA models using heteroscedasticity-robust standard errors showed the most precise effect for CMJ (*p* = 0.020), with V4 (*p* = 0.066) and vIFT (*p* = 0.054, favoring controls) showing smaller effects and IMTP showing no between-group difference (*p* = 0.490). None of these effects survived correction for multiple comparisons (adjusted *p* = 0.078-0.490). This CMJ effect was itself baseline-dependent, negligible at low baseline values and more pronounced at higher ones. For descriptive comparison, the intervention group improved significantly in V4, CMJ, and IMTP (all *p* ≤ 0.05), while controls showed no significant pre-post changes in any outcome. Exploratory individual responder analyses showed the highest proportion of improvers for V4 and IMTP, followed by CMJ.

**Discussion:**

QT proved feasible to implement as an individualized training framework, and effect sizes for V4 and CMJ suggested a beneficial impact on submaximal endurance capacity and explosive lower-body power. These preliminary findings warrant confirmation in adequately powered trials.

## 1 Introduction

Youth soccer matches typically last 2 × 35-45 min and are characterized by intermittent activity patterns, with players repeatedly alternating between low-intensity locomotion, high-speed running, very-high-speed running, and sprinting, continuously challenging multiple physiological systems (1–3). Depending on the position during match play, players cover substantial distances at different running speeds and sprinting intensities and perform numerous short, intense actions, including accelerations, decelerations, changes of direction, jumps, tackles, and repeated sprints (1, 4–7). This repeated transition between low- and high-intensity activity requires the continuous interaction of aerobic and anaerobic energy systems (8, 9), while neuromuscular capacities such as maximal strength, explosive power, and strength endurance are essential for sprinting speed and execution of high-intensity actions throughout the match (10, 11).

From a conditioning perspective, improving endurance in soccer performance depends on both maximal aerobic capacity and submaximal metabolic efficiency (12–14). Maximal aerobic capacity, commonly expressed as VO₂max, supports repeated high-intensity efforts, recovery between sprints, and the maintenance of work rate across the match (15–17). However, because players spend large portions of the game and training at low to moderate intensities, submaximal metabolic efficiency is equally important for sustaining overall match performance (12, 14). Players who cover a given distance at lower metabolic cost may recover more effectively between high-intensity actions and better preserve their capacity to execute them late in the match (18).

Although VO₂max is the criterion measure of maximal aerobic capacity, laboratory assessment is often impractical in lower-ranked clubs and youth settings due to costs, equipment, personnel, and logistical demands (19, 20). Field-based alternatives have therefore been established to enable practical, targeted decision-making in applied soccer contexts (19). Among these, the 30-15 Intermittent Fitness Test (30-15IFT) has gained particular traction, yielding the maximal intermittent running velocity (vIFT), which demonstrates strong associations with both VO₂max and sport-specific intermittent fitness (21–23).

In German soccer diagnostics, outdoor incremental field tests with capillary blood lactate sampling are widely used to determine threshold-related running velocities and to track longitudinal shifts in these parameters (24–26). In particular, the running velocity at a blood lactate concentration of 4 mmol·L⁻¹ (V4) is frequently used as a practical marker of blood lactate accumulation and, consequently, of submaximal endurance performance. Although fixed lactate thresholds have methodological limitations, V4 retains applied utility due to its simplicity, interpretability, and sensitivity to training-induced adaptations (24, 27–30).

A similar logic applies to strength diagnostics. Soccer-specific actions such as sprint acceleration, jumping, tackling and rapid changes of direction require both high force production and rapid force expression (10, 11, 31). Maximal strength and explosive power are related but distinct neuromuscular qualities and may therefore require different training emphases (32). In applied settings, the Isometric Mid-Thigh Pull (IMTP) represents a valid, reliable, and low-risk method for assessing relative maximal force production in athletes (33–35), while countermovement jump height (CMJ) is among the most widely used and validated measures of explosive lower-body power (36–38). Together, IMTP and CMJ offer complementary insight into a player's maximal strength and explosive power.

After data collection, individual endurance and strength profiles can be used to guide the subsequent prescription of targeted training methods. Moderate-intensity continuous training (MICT), performed at approximately 50%–80% of VO₂max over prolonged durations (39, 40), consistently improves submaximal threshold parameters such as V2 (running velocity at a blood lactate concentration of 2 mmol·L⁻¹) and V4, and may therefore be preferentially indicated for players with lower metabolic efficiency (41–43).

High-intensity interval training (HIIT), performed at approximately 80%–170% of VO₂max with shorter exercise duration (39), is particularly effective for improving VO₂max (40, 44–47) and may therefore be preferentially indicated for players with lower maximal aerobic capacity. For trained athletes, strength improvements are maximized through high-load resistance training (>60%–80% 1RM), particularly when sets are taken close to momentary failure (48–51). For the development of explosive power, plyometric and jump training have consistently shown efficacy in youth soccer players, encompassing exercises that exploit the stretch–shortening cycle across both short (<250 ms) and long (>250 ms) ground contact times and typically performed at low external loads to maximize power output (6, 52–54).

The individualized development of endurance and strength capacities represents a central objective in soccer training from both the athlete's and the coach's perspective. Ideally, performance-limiting factors would be identified and addressed through fully individualized training interventions (55). In practice and particularly in youth and lower-ranked settings, such approaches are constrained by limited staff, time, and infrastructure. Given squad sizes of 20 or more players, fully individualized programming is rarely feasible in daily practice (55, 56). Consequently, comparing athletes to the group average, as well as to the highest- and lowest-performing players within the squad, represents a common and practically relevant approach for conditioning-related decision-making and training prescription (57–59).

A practical alternative is to classify players into typological groups based on shared performance characteristics. In the present context, this classification is achieved using a typology-based training allocation into four quadrants followed by typology-specific interventions, an approach we define as “Quadrant Training” (QT). Two related yet distinct performance variables are plotted against each other relative to the team mean, which serves as a reference threshold distinguishing relatively high from relatively low values. This yields four performance typologies: players above average in both variables, below average in both, or above average in one and below average in the other. This approach could be used to assess multiple performance domains simultaneously, thereby creating a comprehensive and multifaceted performance profile.

Applied to endurance, each player's vIFT and V4 values are plotted against the respective team mean, classifying players by relative maximal intermittent fitness and submaximal metabolic efficiency. This classification directly informs training allocation: e.g., low vIFT indicates a primary need for HIIT, low V4 for MICT, and below-average scores on both for a combined approach alternating low- and high-intensity endurance work from week to week. The same logic applies to strength: low IMTP suggests prioritizing heavy resistance training, low CMJ explosive or plyometric training, and below-average scores on both a combined program targeting maximal strength and explosive power.

Thus, QT provides a practical compromise between fully individualized training and uniform team-based training, enabling coaches to target relevant performance deficits while keeping the number of distinct training plans manageable. This approach offers a practical decision-making framework particularly suited for youth soccer settings, where physical development is heterogeneous and resources for individualized programming are often limited. Instead of prescribing one uniform training program for all players or developing separate programs for every individual, coaches can assign players to a limited number of targeted training groups. The approach retains individualized logic since allocation is based on each player's relative performance profile while remaining feasible by limiting the number of distinct programs required and affording coaches clearer oversight and control within each subgroup.

Whether typology-based allocation can effectively guide supplementary training in youth soccer remains unclear. While individual components of endurance and strength training are reasonably well understood, evidence is lacking on whether classifying players according to combined endurance-strength profiles and aligning training content accordingly improves the targeted performance variables. Ideally, such an approach would produce measurable improvements in the specific performance variables targeted by each player's baseline classification.

The first aim of this study was therefore to classify youth soccer players into four typological groups based on key endurance and strength characteristics: endurance typologies derived from vIFT and V4 as indicators of maximal intermittent fitness and submaximal metabolic efficiency, and strength typologies derived from relative IMTP and CMJ as indicators of maximal strength and explosive lower-body power. The second aim was to examine whether QT improves these endurance- and strength-related variables beyond the effects of regular soccer training alone.

We hypothesized that QT would produce greater improvements in V4, vIFT, CMJ, and IMTP compared with regular soccer training alone, with the largest improvements occurring in the specific performance variable(s) targeted by each player's baseline quadrant classification.

## 2 Materials and methods

### 2.1 Experimental approach to the problem

This non-randomized, academy-level controlled intervention study examined endurance- and strength-related adaptations associated with the implementation of Quadrant Training (QT) in youth soccer players. Two German youth performance academies were recruited based on availability and willingness to participate. Both academies competed in the same league. Group allocation was determined at academy level before study commencement and prior to the collection of outcome data. The academy providing the larger available player cohort was assigned to the QT intervention condition, whereas the second academy served as the comparison condition and continued its regular training. No individual- or academy-level randomization was performed. Because only one academy represented each condition, academy and intervention condition were fully confounded and cannot be statistically separated.

The primary comparison was between the two academy-level conditions: the academy implementing QT and the academy continuing regular soccer training. Within the intervention academy, supplementary training content was determined by each player's baseline quadrant classification. Dependent variables comprised vIFT and V4 as indicators of maximal intermittent fitness and submaximal metabolic efficiency, and relative IMTP and CMJ as indicators of maximal strength and explosive lower-body power. These variables were selected because they reflect complementary endurance and neuromuscular qualities central to soccer performance and are amenable to field-based assessment.

### 2.2 Participants

The study involving human participants was conducted in accordance with the Declaration of Helsinki (2013) and approved by the Scientific and Ethical Review Board of the Julius-Maximilians-University Würzburg (EV2025/8-0112). All players and their legal guardians were informed about the study procedures and potential risks prior to participation. Because all participants were under the age of 18 years, written informed consent was obtained from their legal guardians and also from all players.

A total of 60 male youth soccer players (U16–U17; age range: 14–16 years) were enrolled, comprising 38 players in the intervention group and 22 in the control group.

### 2.3 Procedures

Performance diagnostics were conducted at three time points across the competitive season: pre-intervention (June 2025), mid-intervention (August 2025), and post-intervention (November 2025). Testing days relative to training and match schedule were kept consistently within each academy. At each time point, a standardized two-day test battery assessed anthropometric characteristics, endurance capacity, and neuromuscular performance. Testing sessions were scheduled at consistent times of day, and players were instructed to avoid strenuous exercise in the 24 h preceding each session. Not all players were able to complete every assessment at each testing session. Missing observations occurred for practical and health-related reasons, including illness, physical complaints, absence, late arrival, or inability to complete a specific test. Consequently, the number of available observations differed across variables and time points.

#### 2.3.1 Anthropometric measurements

Body height was assessed using a wall-mounted measuring scale, and body mass was measured with a digital body composition scale (Renpho body scale ES-CS20M, Renpho Health, China). In addition, age was recorded. Due to practical constraints during field-based testing, anthropometric measurements were not available for every player at each assessment, and the number of available observations therefore differed between variables. Chronological age at post-intervention was derived from baseline age and the elapsed time between assessments rather than treated as an independently measured longitudinal outcome.

#### 2.3.2 Endurance performance testing

The 30-15 Intermittent Fitness Test (30-15IFT) is a shuttle-run protocol consisting of repeated 30-second running bouts on a 40-m course, separated by 15-second passive recovery periods, with running speed dictated by audio beeps. Initial speed is set at 8 km·h⁻¹ and increases by 0.5 km·h⁻¹ after each 30-second stage. The test terminates at volitional exhaustion or when a participant fails to reach the marked lines (within 3 m) on three consecutive occasions. vIFT corresponds to the running velocity of the last fully completed stage (60).

Submaximal endurance capacity was assessed via an incremental lactate threshold test conducted on a 400-m track. Cones were placed at 50-m intervals and running speed was controlled using an audio pacing signal. Following measurement of resting blood lactate, participants completed successive 1,200-m stages beginning at 2.5 m·s⁻¹, with speed incrementing by 0.5 m·s⁻¹ per stage, except for the final stage (stage 5), which comprised 1,600 m. Between stages, a standardized 120-s recovery interval was provided during which capillary blood samples were obtained from the earlobe for lactate analysis. The test was terminated upon completion of stage 5 or volitional exhaustion. Blood lactate was analyzed using EKF Biosen C-Line. The running velocity at a blood lactate concentration of 4 mmol·L⁻¹ (V4) was subsequently derived via linear interpolation of the individual lactate–velocity relationship.

#### 2.3.3 Strength performance testing

Prior to strength testing, all participants completed a standardized warm-up lasting approximately 10 min incorporating aerobic activation, mobilization and plyometrics. For the IMTP, participants adopted a hip-width stance while knee and hip joint angles were standardized using a goniometer (knee angle: 130 ± 10°; hip angle: 140 ± 10°) (34). The barbell was positioned at mid-thigh level, and participants maintained an upright torso with a neutral spine throughout the test.

Before maximal testing, two familiarization trials were performed at approximately 60% and 80% of perceived maximal effort. Participants then performed maximal upward isometric pulls against the fixed barbell for 3–5 s without countermovement, using a gradual, controlled build-up of force rather than an explosive pull; this instruction was chosen deliberately to avoid transient force overshoot artifacts and to ensure that the recorded peak reflected a stable, sustained maximal force rather than a brief momentary spike. Two maximal trials were completed, with 2–3 min of passive recovery provided between attempts. The primary outcome measure was peak force relative to body mass (N·kg⁻¹).

Before the CMJ test, participants were instructed to stand upright with their feet shoulder-width apart and their hands placed on their hips to minimize variations in jump technique (61). Participants then performed a rapid downward movement to approximately 90° of knee flexion, immediately followed by an explosive upward extension of the lower limbs in order to achieve maximal jump height (countermovement jump). Prior to the recorded trials, two familiarization attempts were completed at approximately 60% and 80% of perceived maximal effort. Subsequently, participants performed three maximal trials, separated by 30–60 s of passive recovery. Jump height was considered the primary performance variable and was used to determine the best trial.

Ground reaction force data were collected using two PASPORT Force Platforms (PS-2142; PASCO Scientific, Roseville, CA, USA) interfaced with PASCO Capstone software at a sampling frequency of 1,000 Hz for all measurements.

### 2.4 Quadrant training (QT) approach

Players in the intervention group were classified into four typology-based quadrants based on their relative team performance in endurance and strength variables (Figure 1). For endurance, classification was based on vIFT and V4. For strength, classification was based on IMTP and CMJ. In both domains, the team mean of each variable was used as the reference value to distinguish between relatively high and low performance.

**Figure 1:**
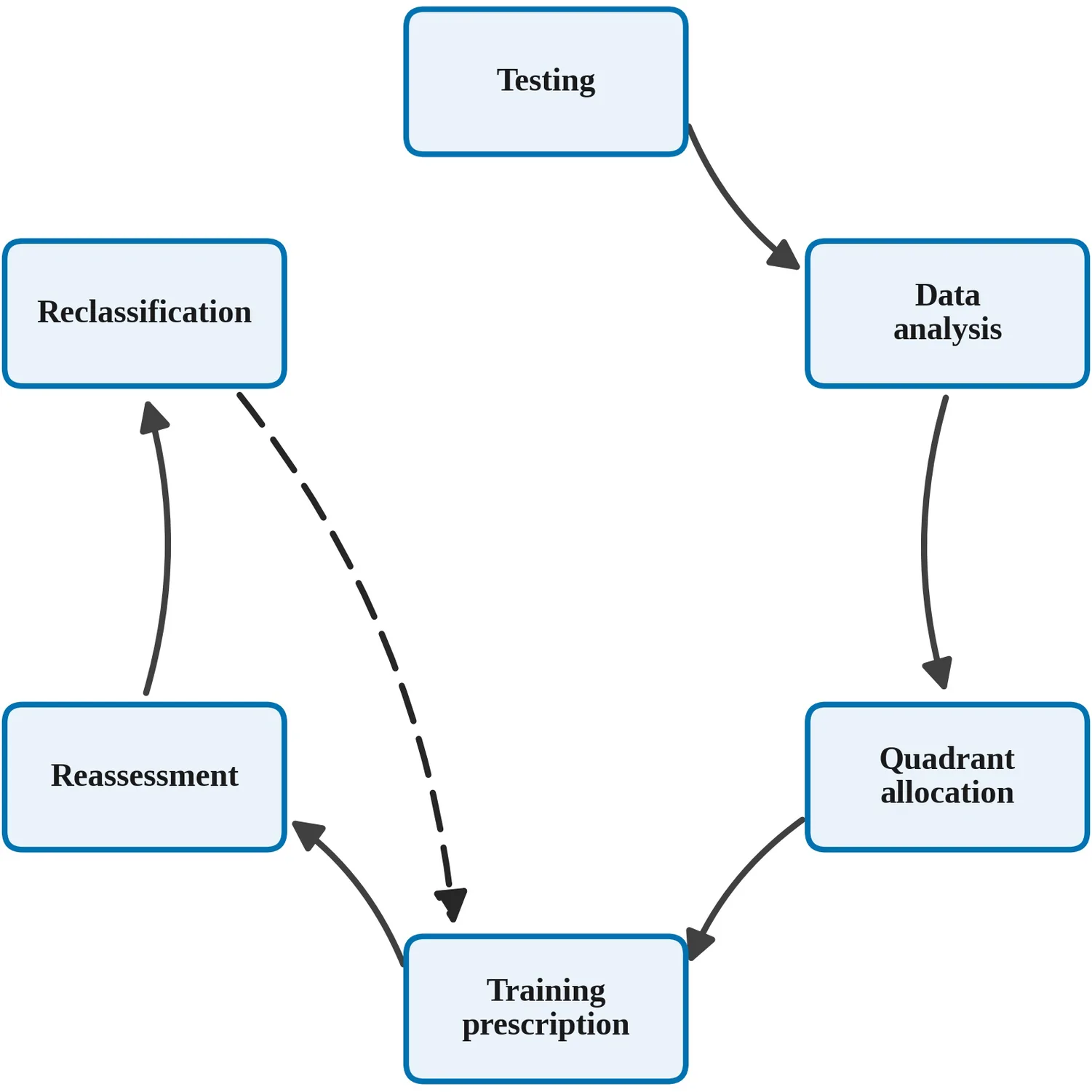
Schematic overview of the quadrant training (QT) process. Following baseline testing, performance data are analyzed relative to the team average, and players are allocated to one of four training quadrants per domain (endurance, strength). Individualized training is prescribed accordingly. Players are subsequently reassessed and reclassified (not done in this study), with reclassification directly informing the training prescription for the next cycle (dashed arrow), reflecting the repeated nature of the QT process.

Based on this classification, players performed typology-specific supplemental training consisting of one additional weekly strength session and one to two endurance sessions targeting their main performance deficits. During the first half of the intervention period (preparation period), two supplementary endurance sessions per week were completed, whereas during the second half of the intervention period (in-season) the frequency was reduced to one supplementary endurance session per week. Depending on the assigned typological quadrant, players then performed either MICT, HIIT, or a combination of both training formats, alternating between MICT and HIIT from week to week. Both formats were matched for total internal training load using the training impulse (Edward's TRIMP) method, which quantifies load as the accumulated duration within each heart-rate intensity zone weighted according to the corresponding exercise-intensity coefficient (62). The HIIT protocol consisted of 4 × 3.5 min intervals performed in heart rate zone 5 [90%–95% of heart rate maximum (HRmax)], interspersed with 5 × 2 min active recovery periods in zone 2 (∼60% HRmax), resulting in a total internal load of approximately 90 TRIMP. The MICT protocol consisted of 36 min of continuous running performed in zone 2.5 (65%–75% HRmax), likewise corresponding to approximately 90 TRIMP. Half-zones (e.g., zone 2.5) were used where the prescribed training intensity fell between two standard Edward's TRIMP zone boundaries, in order to more precisely reflect the intended intensity rather than rounding to the nearest whole zone.

**Quadrant 1** comprised players with above-average vIFT and V4 values, who received a combined endurance stimulus including both MICT and HIIT to maintain and further develop both maximal intermittent fitness and submaximal aerobic performance. **Quadrant 2** included players with below-average vIFT but above-average V4 values; therefore, training primarily emphasized HIIT to improve maximal intermittent fitness. **Quadrant 3** consisted of players with below-average values in both vIFT and V4, who completed a combined endurance approach including both HIIT and MICT to improve both performance domains simultaneously. **Quadrant 4** included players with above-average vIFT but below-average V4 values and therefore primarily performed MICT to target submaximal aerobic performance.

Depending on the assigned typological quadrant, training intervention consisted of heavy resistance training, explosive/plyometric training, or a combination of both. Heavy resistance exercises were typically performed at intensities >80%-85% of one-repetition maximum (1RM) for 3-4 repetitions per set and 3-4 sets per exercise. Explosive and plyometric exercises were performed with maximal voluntary movement velocity for 4-6 repetitions per set and 3-4 sets per exercise. Plyometric exercises included squat jumps, countermovement jumps, trap bar jumps (20%-30% 1RM), box jumps, and drop jumps, whereas heavy resistance exercises included back squats, Romanian deadlifts, split squats, trap bar squats, and hip thrusts.

**Quadrant 1** comprised players with above-average maximal strength and explosive power values. These players completed a combined maintenance-oriented strength stimulus including both explosive and heavy resistance exercises to maintain maximal and explosive force production. **Quadrant 2** included players with above-average explosive power but below-average maximal strength values. Therefore, training primarily emphasized heavy resistance training to improve maximal strength, supplemented by a small volume of explosive exercises to maintain explosive force production. **Quadrant 3** consisted of players with below-average values in both maximal strength and explosive power. These players completed a combined strength approach with higher overall training volume targeting improvements in both maximal and explosive force production. **Quadrant 4** included players with above-average maximal strength but below-average explosive power values. Training therefore primarily emphasized explosive and plyometric exercises to improve explosive force production, supplemented by a small volume of heavy resistance training to maintain maximal strength.

Quadrant classification was performed once at baseline (PRE) and held fixed for the full intervention period; players were not reclassified or reassigned to a different training emphasis following the mid-intervention (MID) assessment. MID testing served solely to monitor trajectory and inform the missing-data approach and was not used to trigger reclassification during the present study.

Adverse events and training-related complaints during the intervention period were not systematically recorded by the research team; player welfare and any training-related concerns were monitored and managed internally by each academy's coaching staff. No intervention-related dropouts or injuries were reported to the research team, and no injury events occurred during testing sessions. We note the absence of independent, systematic adverse-event monitoring as a limitation of the present study.

### 2.5 Control group training

The control group continued its regular soccer training, consisting of four to five soccer-specific sessions lasting approximately 60–90 min and one match per week. The regular soccer-specific training and match schedules of both academies were comparable in terms of frequency and duration. However, total training exposure was not matched between conditions because the intervention academy additionally completed the supplementary QT-specific strength and endurance sessions described above. Players in the control academy occasionally performed body-weight-based strength exercises approximately every one to two weeks, but no QT or comparable typology-based training allocation was implemented.

### 2.6 Statistical analyses

Missing data were handled using multiple imputation with a multivariate imputation by chained equations approach (63), and parameter estimates were pooled according to Rubin's rules with the Barnard-Rubin degrees-of-freedom correction, which is recommended over the classical Rubin (1987) formula for the sample sizes in the present study (64, 65). Prior to imputation, the missing-data mechanism was examined. Missingness in post-intervention values was not significantly associated with baseline performance (*p* = 0.08–0.58 across outcomes) or group (*p* ≥ 0.29), providing no evidence of differential dropout and supporting a missing-at-random mechanism. Imputation was performed in wide format, with baseline, mid-intervention, and post-intervention values for all four outcomes (V4, vIFT, CMJ, IMTP) included jointly in a single cross-outcome imputation model, estimated separately by group to avoid information leakage between the intervention and control conditions. This joint, cross-outcome specification was adopted as the primary approach because it allows correlated information from all measured outcomes and timepoints to inform each imputed value; narrower, outcome-specific and domain-grouped imputation specifications were evaluated as sensitivity analyses and produced directionally and qualitatively consistent results (Section 3.4; Supplementary Table S1), supporting the robustness of the primary specification. V4 was imputed directly rather than reconstructed from the underlying lactate–velocity curve, to avoid altering observed, non-imputed values through *post-hoc* smoothing. For a given outcome, a player's data were treated as missing only if values were unavailable at all three testing occasions (PRE, MID, and POST); imputed values falling outside this criterion were masked to missing after the imputation model was estimated, rather than restricted during imputation itself, to avoid destabilizing the predictor matrix for correlated outcomes. Mid-intervention values served as an auxiliary predictor to improve imputation accuracy but were not analyzed as a primary outcome. For descriptive completeness, observed MID values (intervention vs. control) were: V4 4.11 ± 0.22 vs. 3.79 ± 0.37 m·s⁻¹; vIFT 20.50 ± 0.82 vs. 21.07 ± 0.94 km·h⁻¹; CMJ 36.04 ± 4.50 vs. 35.18 ± 3.80 cm; IMTP 19.50 ± 3.11 vs. 17.95 ± 3.32 N·kg⁻¹. Missing-data patterns and imputed values were inspected for plausibility. Prior to inferential analyses, model assumptions were checked; for ANCOVA models, residual diagnostics, variance homogeneity, linearity between baseline and post-intervention values, and homogeneity of regression slopes were examined. These diagnostics identified relevant heteroscedasticity for several outcomes and a significant baseline-by-group interaction for CMJ (Section 3.3.1), which informed the use of robust standard errors and simple-slope analyses described below. Additionally, sensitivity analyses were conducted using the non-imputed complete-case dataset, including within-group paired t-tests and descriptive comparisons of pre-post changes between the observed and imputed datasets, to assess whether the primary imputation approach substantially altered the overall pattern of results.

An *a priori* power analysis was conducted based on an independent-samples t-test, a planning effect size of d = 0.82, a statistical power of 80%, and a Bonferroni-adjusted significance level of *α* = 0.0125 to account for the four primary outcomes, yielding a required sample size of approximately 35 players per group (70 in total).

Statistical analyses were performed to compare post-intervention outcomes between the intervention group and the control group. The primary outcomes were V4, vIFT, CMJ and IMTP. For each outcome, an analysis of covariance (ANCOVA) model was applied with post-intervention values as the dependent variable, baseline performance as a covariate, and group as the independent variable.

Because preliminary diagnostic checks indicated relevant heteroscedasticity in a substantial proportion of imputed datasets for several outcomes (Section 3.3.1), heteroscedasticity-consistent (HC3) standard errors were used as the primary basis for inference for all between-group ANCOVA effects; conventional, model-based standard errors are reported for comparison in Supplementary Table S1a. To account for testing four primary outcomes, *p*-values for the between-group effects were additionally adjusted for multiple comparisons using the Benjamini-Hochberg procedure, and adjusted *p*-values are reported alongside nominal *p*-values throughout. Where the assumption of homogeneous regression slopes was violated, an interaction term between baseline value and group was added to the corresponding ANCOVA model, and simple-slope estimates were derived at the 25th, 50th, and 75th percentiles of the baseline distribution to characterize how the group effect varied with baseline performance. As sensitivity checks, the primary between-group ANCOVA models were additionally re-estimated (a) using multiple imputation restricted to players with observed data at all three testing time points (Supplementary Table S1b) and (b) using the non-imputed complete-case dataset (Supplementary Table S1c).

To support individual interpretation of pre-to-post changes, percentage changes [(POST−PRE)/PRE×100] were evaluated against test-specific reliability thresholds derived from published test-retest data in athletic and team-sport populations. The coefficient of variation (CV%) served as the primary indicator of measurement variability, given that all individual changes were expressed relative to baseline. Changes not exceeding the respective CV% were interpreted as falling within normal test-retest variation; changes exceeding it were considered likely to reflect a true performance change. Based on published reliability data, the following thresholds were applied: 5% for vIFT (corresponding to approximately one meaningful increment of ∼1.0 km·h⁻¹) (22, 66); 5% for V4 (reflecting protocol- and analyzer-dependent variability inherent to lactate-derived thresholds) (24, 28); 5% for CMJ (37); and 7% for IMTP (34, 67). Primary analyses were conducted across imputed datasets, with mean percentage changes, standard errors, 95% confidence intervals, and *p*-values pooled according to Rubin's Rules. Because responder classification was performed within each of the 50 imputed datasets and then averaged, individual-level responder counts are generally non-integer; this averaging procedure was verified by cross-checking pooled percentages against the underlying per-imputation classification counts for a subset of outcomes. For the primary performance outcomes (V4, vIFT, CMJ, and IMTP), conspicuous values were reviewed for plausibility and retained unless they represented clear data-entry errors. Anthropometric variables were evaluated separately for descriptive reporting; following re-evaluation, implausible longitudinal changes in body height were not interpreted as physiological adaptations, and height is therefore reported at baseline only. Chronological age was derived from baseline age and the elapsed follow-up time and was reported descriptively. This approach complemented the descriptive presentation of percentage changes and was employed to distinguish training-induced adaptations from changes attributable to normal biological and technical measurement error.

In addition, an exploratory quadrant-specific analysis was conducted within the intervention group. Individual percentage-change plots were supplemented by indicating each player's baseline quadrant allocation, allowing player-level responses to be visually interpreted according to the initial performance profile. Furthermore, to examine whether the observed adaptations were descriptively consistent with the intended quadrant-specific training emphasis and led to the intended improvement in the corresponding variables, pooled percentage changes with 95% confidence intervals and CV-based responder classifications were summarized within each baseline quadrant.

Effect sizes were calculated using partial eta squared (*η*²ₚ) and interpreted as small (0.01), medium (0.06), and large (0.14). Under multiple imputation, *η*²ₚ and its confidence interval were computed separately within each of the 50 imputed datasets, using the robust (HC3) standard errors and Barnard-Rubin-adjusted degrees of freedom for that imputation, and the resulting values were then averaged arithmetically across imputations; this averaging approach is a commonly used approximation rather than a formally derived pooling rule, as *η*²ₚ does not follow a distribution for which Rubin's rules were originally derived. This procedure explains why the reported *η*²ₚ values are smaller than a naive back-calculation from the pooled t/*p*-values would suggest, since the latter would implicitly assume the unadjusted, larger classical degrees of freedom. Results are reported with 95% confidence intervals. All analyses were conducted in R (R Foundation for Statistical Computing), using the mice package for multiple imputation and the sandwich package for heteroscedasticity-consistent (HC3) standard errors. Statistical significance was set at *p* ≤ 0.05.

## 3 Results

### 3.1 Participant characteristics

Anthropometric characteristics are presented in Table 1. Sample sizes differ between variables because individual anthropometric assessments were not available for all players. Body height is reported at baseline only because the available post-intervention measurements showed implausible longitudinal changes that could not be confidently distinguished from measurement or recording variability. Age is reported descriptively and was updated according to the elapsed time between pre- and post-testing.

**Table 1:** Descriptive statistics for anthropometric characteristics in the intervention and control group.

Anthropometric Characteristics				
Variable	n	Pre-Testing M ± SD	Post-Testing M ± SD	*Δ* M ± SD
Intervention Group				
Height (cm)	33	177.26 ± 7.94	-	-
Weight (kg)	20	65.98 ± 9.30	67.17 ± 7.90	+1.19 ± 2.53*
Age	34	15.21 ± 0.77	15.62 ± 0.77	-
Control Group				
Height (cm)	19	181.03 ± 6.34	-	-
Weight (kg)	10	67.03 ± 7.27	67.76 ± 6.13	+0.73 ± 1.63
Age	19	15.88 ± 0.36	16.30 ± 0.36	-

Values are presented as mean ± standard deviation. Age was calculated based on the time interval between pre-testing in June and post-testing in November and is reported descriptively only. Height is reported at pre-testing only because implausible longitudinal changes suggested measurement variability or recording inconsistencies. Because weight was analyzed via matched pre-to-post comparisons, the reported *n* reflects only players with complete data at both time points. This additional attrition primarily reflects time constraints during the two-day testing battery, as players arriving late or needing to leave early most often missed the weight measurement specifically. Asterisks indicate significant within-group changes from pre- to post-testing (*p* ≤ 0.05).

Sample flow for the four primary outcomes (V4, vIFT, CMJ, IMTP), including the number of observed baseline and post-intervention values, complete cases, and the multiply imputed analytic sample used in the primary analyses, is presented in Table 2.

**Table 2:** Observed baseline performance characteristics and outcome-specific data availability at pre- and post-intervention.

Baseline Performance Characteristics and Data Availability						
Variable	Baseline n	Baseline M ± SD	Post n	Complete-case n	Analytic *n* after MI	Missing (%) Baseline/Post
Intervention Group (enrolled *n* = 38)						
V4 (m·s⁻¹)	31	3.85 ± 0.31	22	18	38	18.4/42.1
vIFT (km·h⁻¹)	32	20.42 ± 0.92	21	18	35	15.8/44.7
CMJ (cm)	33	35.14 ± 3.57	23	20	36	13.2/39.5
IMTP (*N*·kg⁻¹)	33	16.94 ± 2.83	23	20	36	13.2/39.5
Control Group (enrolled *n* = 22)						
V4 (m·s⁻¹)	16	3.74 ± 0.36	12	10	19	27.3/45.5
vIFT (km·h⁻¹)	18	20.64 ± 1.27	8	6	21	18.2/63.6
CMJ (cm)	19	35.19 ± 4.93	11	9	21	13.6/50.0
IMTP (N·kg⁻¹)	18	15.86 ± 2.67	11	8	21	18.2/50.0

Baseline M ± SD values are based on the original observed, non-imputed baseline measurements. Baseline *n* and Post *n* indicate the number of observed measurements at the respective time point. Missing percentages are calculated relative to the number of enrolled players within each group (intervention *n* = 38; control *n* = 22) and are shown as baseline/post-intervention percentages. Complete-case *n* denotes players with both observed baseline and post-intervention values for the respective outcome. Analytic *n* after MI denotes the outcome-specific sample included in the multiply imputed primary analyses; imputed values are not included in the descriptive baseline M ± SD values shown here.

### 3.2 Within-Group analysis

In the within-group analyses, the intervention group demonstrated significant improvements from pre- to post-testing in V4 [*Δ* = 0.246, 95% CI (0.121, 0.371), *p* < 0.001], CMJ [*Δ* = 1.528, 95% CI (0.080, 2.975), *p* = 0.040], and IMTP [*Δ* = 1.068, 95% CI (0.007, 2.129), *p* = 0.049]. No significant change was observed for vIFT [*Δ* = −0.202, 95% CI (−0.541, 0.137), *p* = 0.228].

The control group showed no statistically significant pre-post changes in any outcome, including V4 [*Δ* = 0.119, 95% CI (−0.124, 0.362), *p* = 0.302], vIFT [*Δ* = 0.319, 95% CI (−0.437, 1.074), *p* = 0.369], CMJ [*Δ* = −1.290, 95% CI (−3.653, 1.073), *p* = 0.262], and IMTP [*Δ* = 1.080, 95% CI (−1.118, 3.278), *p* = 0.307].

### 3.3 Between-Group effects (ANCOVA)

Between-group effects were estimated using pooled ANCOVA models on multiply imputed datasets, adjusted for baseline values. Because model diagnostics indicated heteroscedasticity in a substantial proportion of imputed datasets (42%–48% for V4, vIFT, and CMJ), heteroscedasticity-consistent (HC3) standard errors are reported as the primary inference; conventional standard errors are provided in Supplementary Table S1 for comparison. To account for testing four primary outcomes, *p*-values were additionally adjusted using the Benjamini-Hochberg procedure. None of the four between-group effects survived correction for multiple comparisons (adjusted *p* = 0.078–0.490). For V4, the intervention group showed an increase in the imputed data (*Δ* =  + 0.246, *n* = 38), while the control group showed a smaller increase (*Δ* =  + 0.119, *n* = 19). The between-group effect approached but did not reach nominal significance [b = −0.201, robust SE = 0.105, 95% CI (−0.416, 0.014), *p* = 0.066, adjusted *p* = 0.088], with an effect size in the moderate range [*η*²ₚ = 0.125, 95% CI (0.017, 0.286)].

For vIFT, the intervention group showed a slight decrease in the imputed data (*Δ* = −0.202, *n* = 35), whereas the control group demonstrated an increase (*Δ* =  + 0.319, *n* = 21). The between-group effect also approached but did not reach nominal significance [b = 0.620, robust SE = 0.304, 95% CI [−0.013, 1.253], *p* = 0.054, adjusted *p* = 0.088, *η*²ₚ = 0.179, 95% CI [0.043, 0.343]]; the direction of this effect favored the control group. Given the consistency of effect sizes for V4 and vIFT between the primary and sensitivity models (multiple imputation restricted to players with data at all three time points, and complete-case analysis; Supplementary Table S1), these findings are interpreted as suggestive of genuine, if imprecisely estimated, training effects rather than as statistical artifacts, most plausibly reflecting limited statistical power given the available sample size. Full model estimates, including conventional and robust standard errors, are reported in Table 3. Endurance outcomes for the intervention and control groups are shown in Figures 2, 3.

**Table 3:** Between-group effect estimates (robust standard errors), unadjusted and Benjamini-Hochberg-adjusted *p*-values, and partial eta-squared effect sizes for primary outcomes.

Between-Group Effect Estimates and Effect Sizes						
Outcome	b	robust SE	95% CI	*p*	adj. *p* (BH)	*η*²_P_ [95% CI]
V4	−0.201	0.105	[−0.416, 0.014]	0.066	0.088	0.125 [0.017, 0.286]
vIFT	0.620	0.304	[−0.013, 1.253]	0.054	0.088	0.179 [0.043, 0.343]
CMJ	−2.778	1.139	[−5.084, −0.471]	0.020	0.078	0.136 [0.017, 0.300]
IMTP	−0.680	0.975	[−2.662, 1.303]	0.490	0.490	0.024 [0.000, 0.135]

b, unstandardized adjusted effect of the control group relative to the intervention group (reference category) from the ANCOVA model; negative values indicate a comparatively more favorable adjusted outcome in the intervention group, positive values a more favorable adjusted outcome in the control group; robust SE, heteroscedasticity-consistent (HC3) standard error; *p*, nominal *p*-value; adj. *p* (BH), Benjamini-Hochberg-corrected *p*-value across the four primary outcomes; η²ₚ = partial eta-squared with 95% confidence interval. Robust SE and BH-corrected *p*-values represent the primary inference criterion for this study (see Section 2.6); naive (non-robust) standard errors are reported for comparison in Supplementary Table S1. No outcome remained statistically significant after correction for multiplicity (adj. *p* range: 0.078–0.490). CMJ showed the lowest nominal *p*-value and a consistent effect size and is interpreted as the strongest, though not statistically significant, finding.

**Figure 2:**
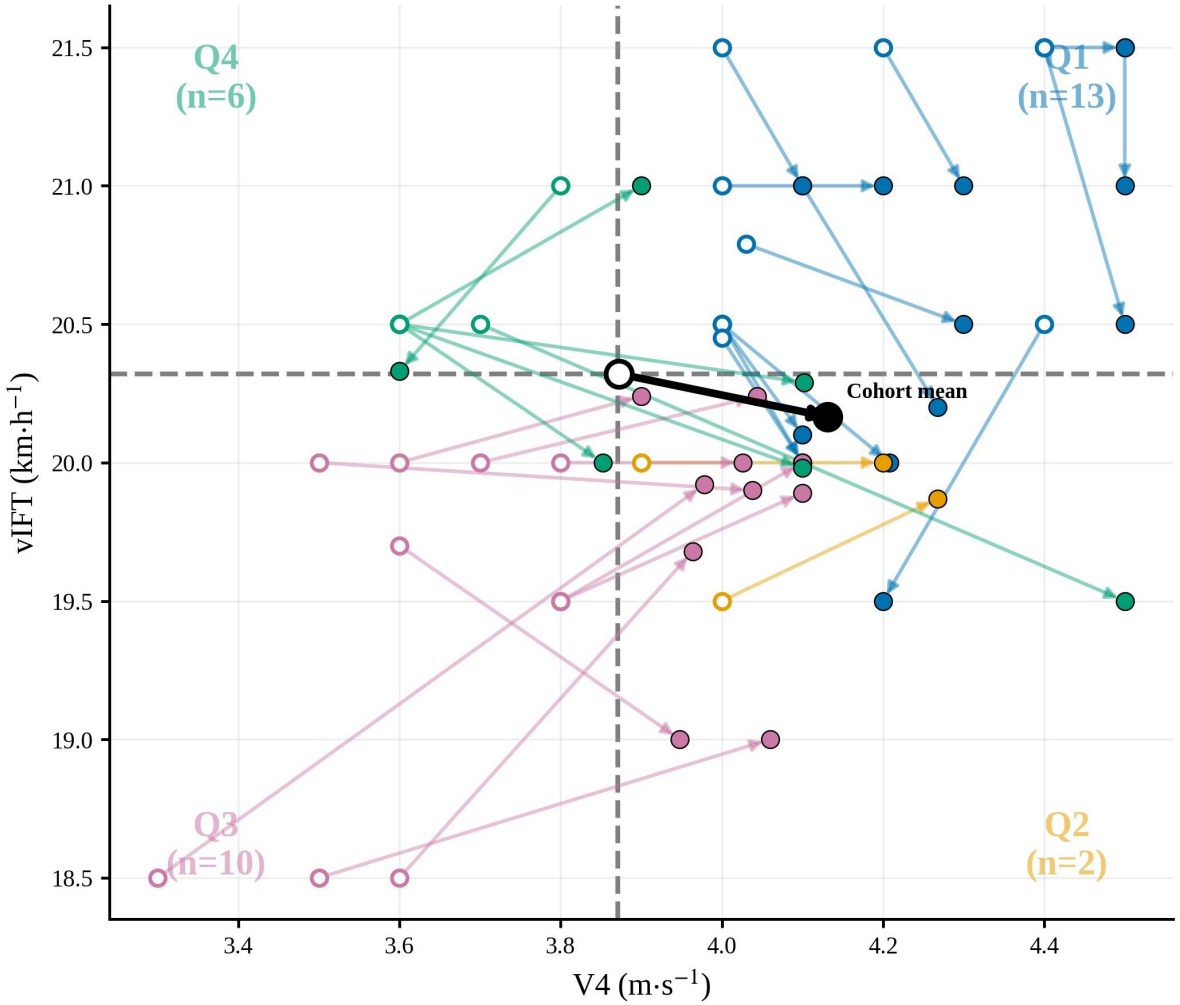
Endurance quadrant classification and individual pre-to-post change in the intervention group (*n* = 31). Hollow circles indicate PRE values, filled circles indicate POST values, thin arrows show individual change trajectories, colored by baseline quadrant (Q1–Q4). The bold black arrow indicates the cohort mean change (black circle = mean POST position). Dashed lines represent the group average (classification threshold). V4: velocity at 4 mmol·L⁻¹ blood lactate; vIFT: maximum velocity in 30-15 Intermittent Fitness Test. Because both V4 and vIFT values are required to plot a given player, the displayed sample (*n* = 31 of 38 enrolled) reflects the intersection of both outcomes' analytic samples and may therefore differ modestly from the outcome-specific between-group estimates reported in the Results section.

**Figure 3:**
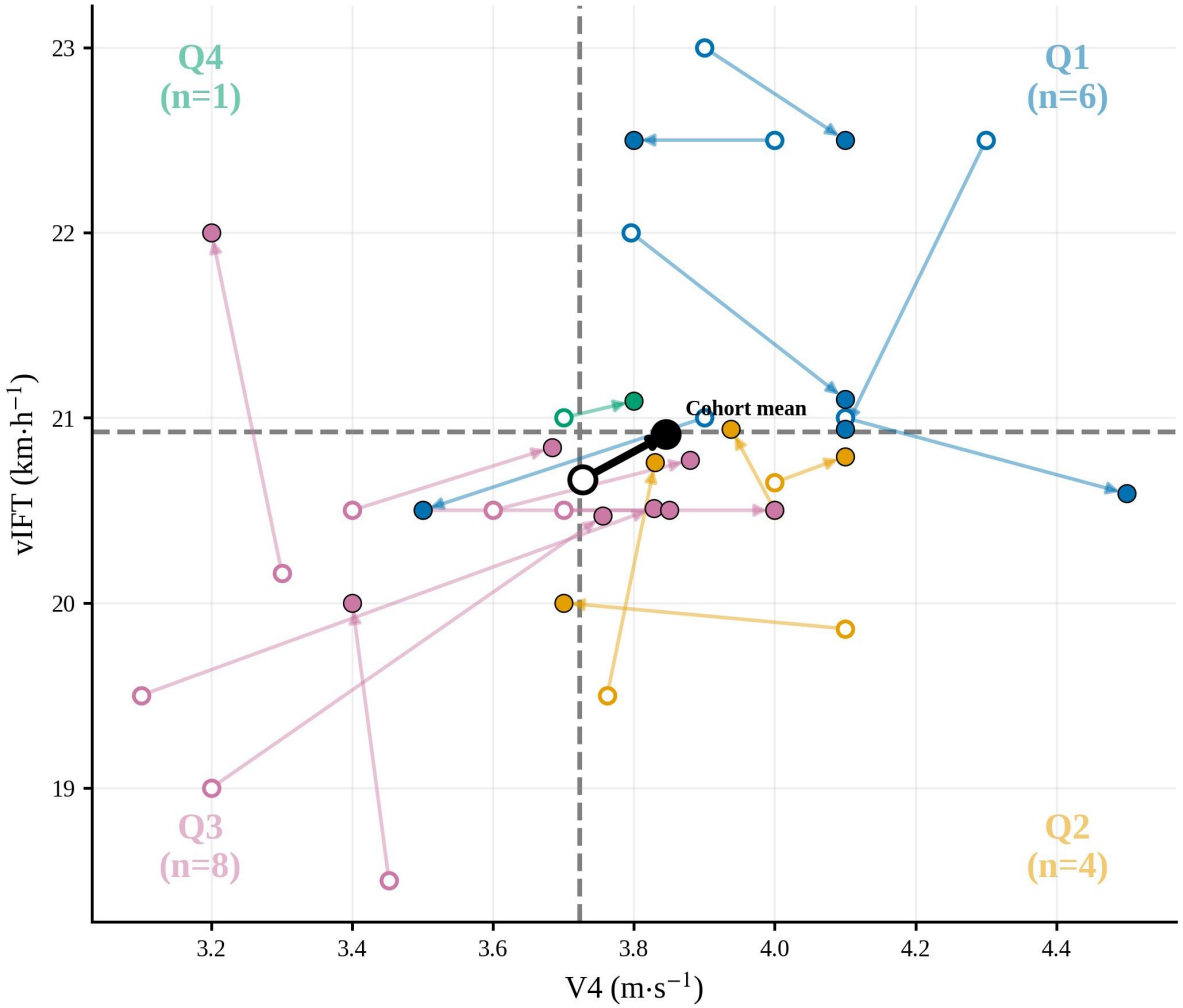
Endurance quadrant classification and individual pre-to-post change in the control group (*n* = 19). Plotting conventions as in Figure 2. Because the control group was not allocated to quadrant-based training, quadrant membership shown here is descriptive only, based on the control group's own baseline mean. The displayed sample (*n* = 19 of 22 enrolled) reflects the intersection of both outcomes' analytic samples.

For CMJ, the intervention group showed an increase in the imputed data (*Δ* = + 1.528 cm, *n* = 36), whereas the control group showed a decrease (*Δ* = −1.290 cm, *n* = 21). CMJ showed the lowest nominal *p*-value among the four outcomes and the most consistent effect size across sensitivity analyses [b = −2.778, robust SE = 1.139, 95% CI [−5.084, −0.471], *p* = 0.020, adjusted *p* = 0.078, *η*²ₚ = 0.136, 95% CI [0.017, 0.300]], although this did not survive correction for multiple comparisons. In addition, a strong association with baseline performance was identified [PRE: b = 0.669, *p* < 0.001, 95% CI (0.351, 0.987)]; however, baseline value significantly interacted with group (Section 3.3.1), indicating this effect was not constant across baseline levels.

For IMTP, the intervention group showed an increase in the imputed data (*Δ* = + 1.068, *n* = 36), while the control group also demonstrated an increase (*Δ* =  + 1.080, *n* = 21). The ANCOVA indicated no evidence of a group effect [b = −0.680, robust SE = 0.975, 95% CI (−2.662, 1.303), *p* = 0.490, adjusted *p* = 0.490], with a small effect size [*η*²ₚ = 0.024, 95% CI (0.000, 0.135)]. Strength outcomes for the intervention and control groups are shown in Figures 4,5.

**Figure 4:**
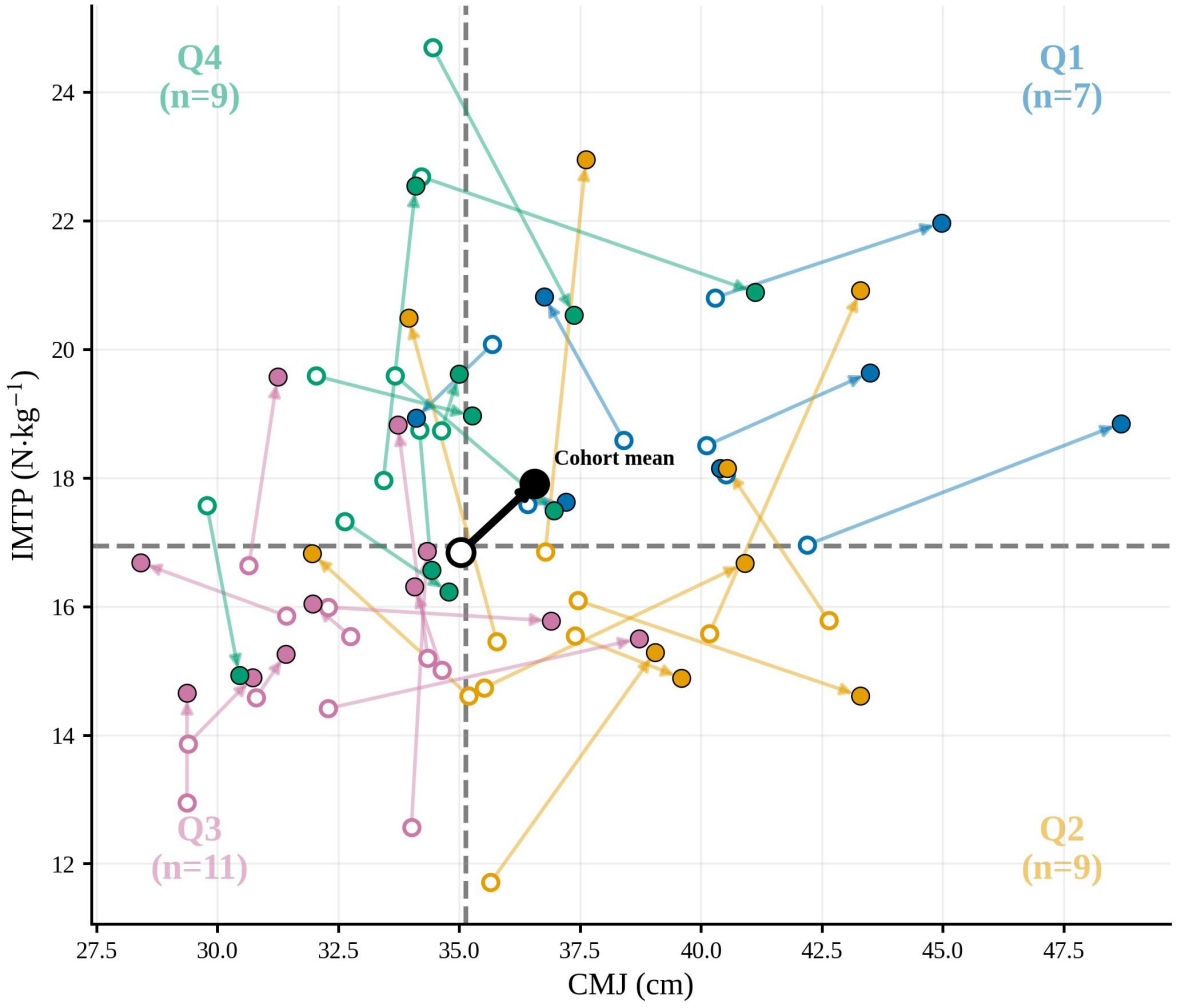
Strength quadrant classification and individual pre-to-post change in the intervention group (*n* = 36). Plotting conventions as in Figure 2. CMJ: countermovement jump height; IMTP: relative isometric mid-thigh pull strength.

**Figure 5:**
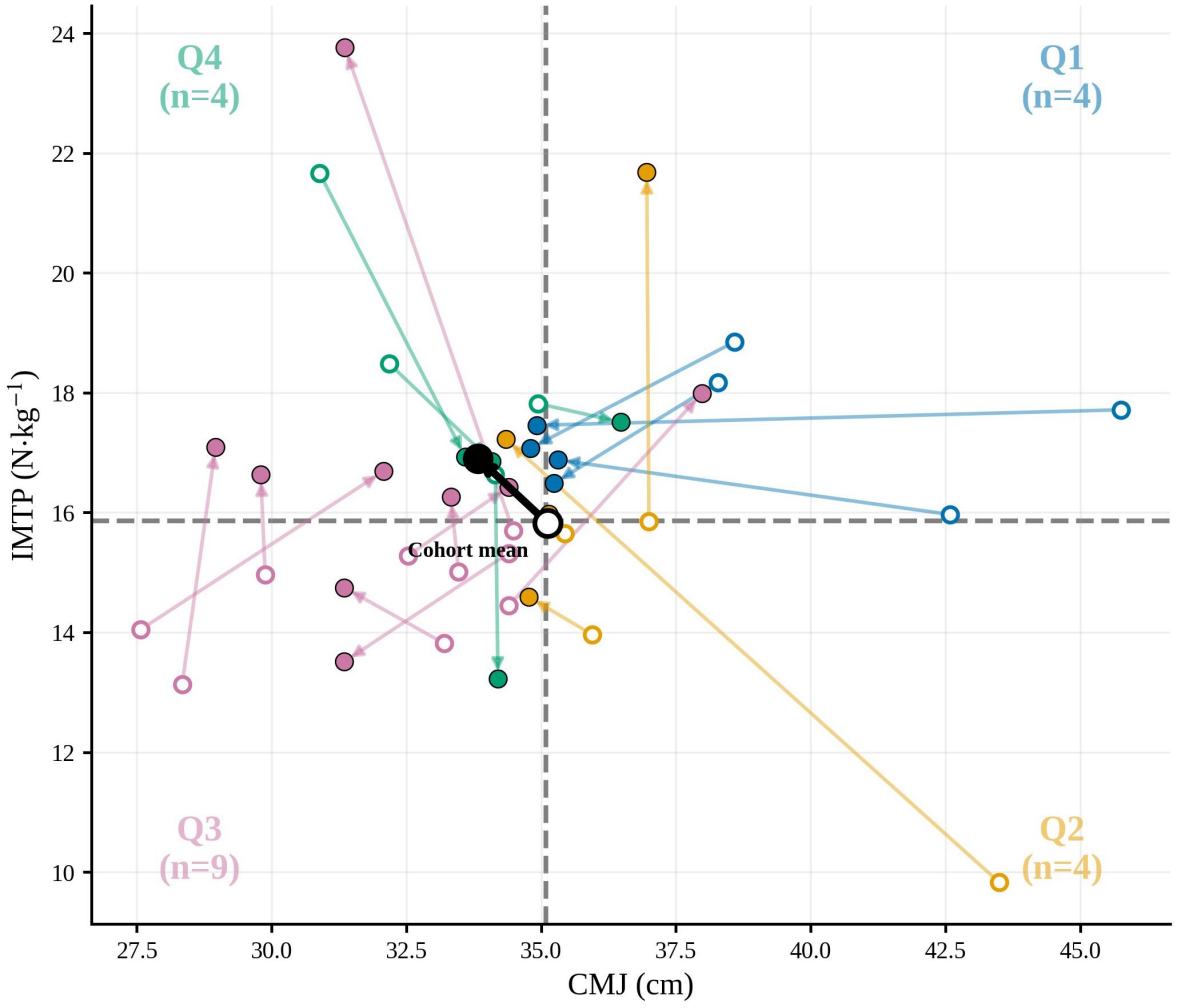
Strength quadrant classification and individual pre-to-post change in the control group (*n* = 21). Plotting conventions as in Figure 2; quadrant membership is descriptive only (see Figure 3).

#### 3.3.1 Model diagnostics

ANCOVA assumption checks indicated no relevant violation of residual normality for V4 (0%) and CMJ (6%), but violations for vIFT (22%) and IMTP (56%). Slope homogeneity held for V4 (2%) and vIFT (2%), but was violated for IMTP (44%) and CMJ (92%, motivating the interaction term below). An analogous PRE × group interaction model for IMTP was non-significant (b = −0.44, SE = 0.50, *p* = 0.39), indicating a genuine null finding rather than a masked baseline-dependent effect. Given the sample size and robust standard errors used throughout, remaining violations are unlikely to materially bias the estimates. For CMJ, the interaction between baseline value and group was significant in the pooled model (b = −0.80, *p* = 0.020), indicating that the group difference was not constant across baseline levels: simple-slope estimates showed a negligible, non-significant group difference at low baseline CMJ (25th percentile: b = −0.68, *p* = 0.61) that increased in magnitude and significance toward higher baseline CMJ (median: b = −2.34, *p* = 0.029; 75th percentile: b = −4.39, *p* < 0.001). Heteroscedasticity was present in 42%-48% of imputations for V4, vIFT, and CMJ, motivating the use of robust standard errors reported above.

### 3.4 Sensitivity analysis for imputed data

Sensitivity analyses based on the non-imputed complete-case dataset generally supported the pattern observed in the imputed analyses. Descriptively, the observed complete-case data and the imputed datasets showed broadly comparable pre–post patterns. In the intervention group, V4, CMJ, and IMTP increased in both datasets, whereas vIFT showed a small decrease in both datasets. In the control group, changes were generally small and the confidence intervals included zero in both the observed and imputed data. The only notable difference was observed for CMJ, where the complete-case data indicated a small positive change, whereas the imputed estimate suggested a negative change. Overall, the sensitivity analyses did not indicate that multiple imputation substantially altered the main descriptive pattern of results. Complete-case paired t-tests showed significant pre-post improvements in the intervention group for V4 [*Δ* =  + 0.183, 95% CI (0.067, 0.299), *p* = 0.00393], CMJ [*Δ* =  + 1.327 cm, 95% CI (0.151, 2.503), *p* = 0.0290], and IMTP [*Δ* =  + 1.884, 95% CI (0.806, 2.962), *p* = 0.00168]. The change in vIFT did not reach the 5% significance level [*Δ* = −0.250, 95% CI (−0.509, 0.009), *p* = 0.0579]. In the control group, no significant pre-post changes were observed for any outcome: V4 [*Δ* =  + 0.000, 95% CI (−0.224, 0.224), *p* = 1.000, *n* = 10], vIFT [*Δ* =  + 0.083, 95% CI (−0.689, 0.856), *p* = 0.793, *n* = 6], CMJ [*Δ* =  + 0.260, 95% CI (−0.952, 1.472), *p* = 0.634, *n* = 9], and IMTP [*Δ* =  + 0.919, 95% CI (−1.013, 2.850), *p* = 0.298, *n* = 8]. Results were additionally consistent in direction and effect size between the primary multiple imputation model and a sensitivity model restricted to players with data available at all three testing time points, as well as between the multiple-imputation and complete-case (non-imputed) analyses (Supplementary Table S1), further supporting the robustness of the reported pattern to the imputation approach chosen.

### 3.5 Pooled percentage changes and CV-based individual responses

The pooled analysis of percentage changes indicated improvements in the intervention group for V4 [+6.75%, 95% CI (3.32, 10.19)], CMJ [+4.36%, 95% CI (0.17, 8.55)], and IMTP [+7.84%, 95% CI (1.48, 14.19)], with 95% confidence intervals excluding zero for all three; vIFT showed no clear change [−0.88%, 95% CI (−2.56, 0.80)]. In the control group, no percentage change estimate excluded zero: V4 [+3.78%, 95% CI (−3.20, 10.76)], vIFT [+1.78%, 95% CI (−1.97, 5.53)], CMJ [−2.46%, 95% CI (−8.59, 3.66)], and IMTP [+9.93%, 95% CI (−6.06, 25.92)]. Figures 6–9 show the individual percentage changes for all parameters for the intervention and control group, relative to coefficient-of-variation (CV) based thresholds for measurement variability.

**Figure 6:**
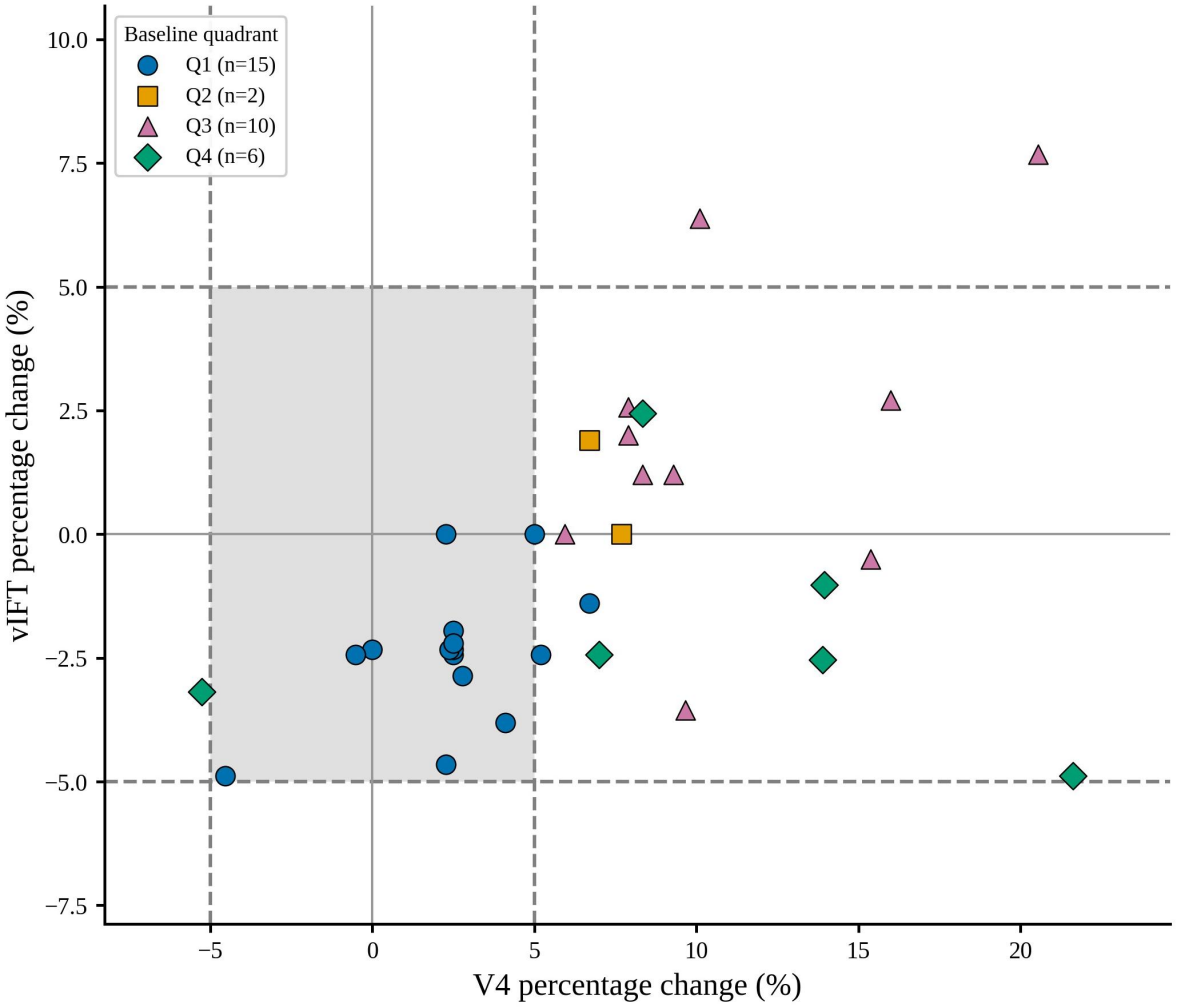
Individual percentage changes in V4 and vIFT relative to coefficient-of-variation (CV) thresholds in the intervention group (*n* = 33), colored and shaped by baseline quadrant. The shaded region indicates change within the CV threshold (±5%); points outside this region exceed typical measurement variability. V4: velocity at 4 mmol·L⁻¹ blood lactate; vIFT: maximum velocity in 30-15 Intermittent Fitness Test. *n* = 33 of 38 enrolled reflects the intersection of both outcomes' analytic samples; unlike Figure 2, no additional visual-consistency filtering applies here.

**Figure 7:**
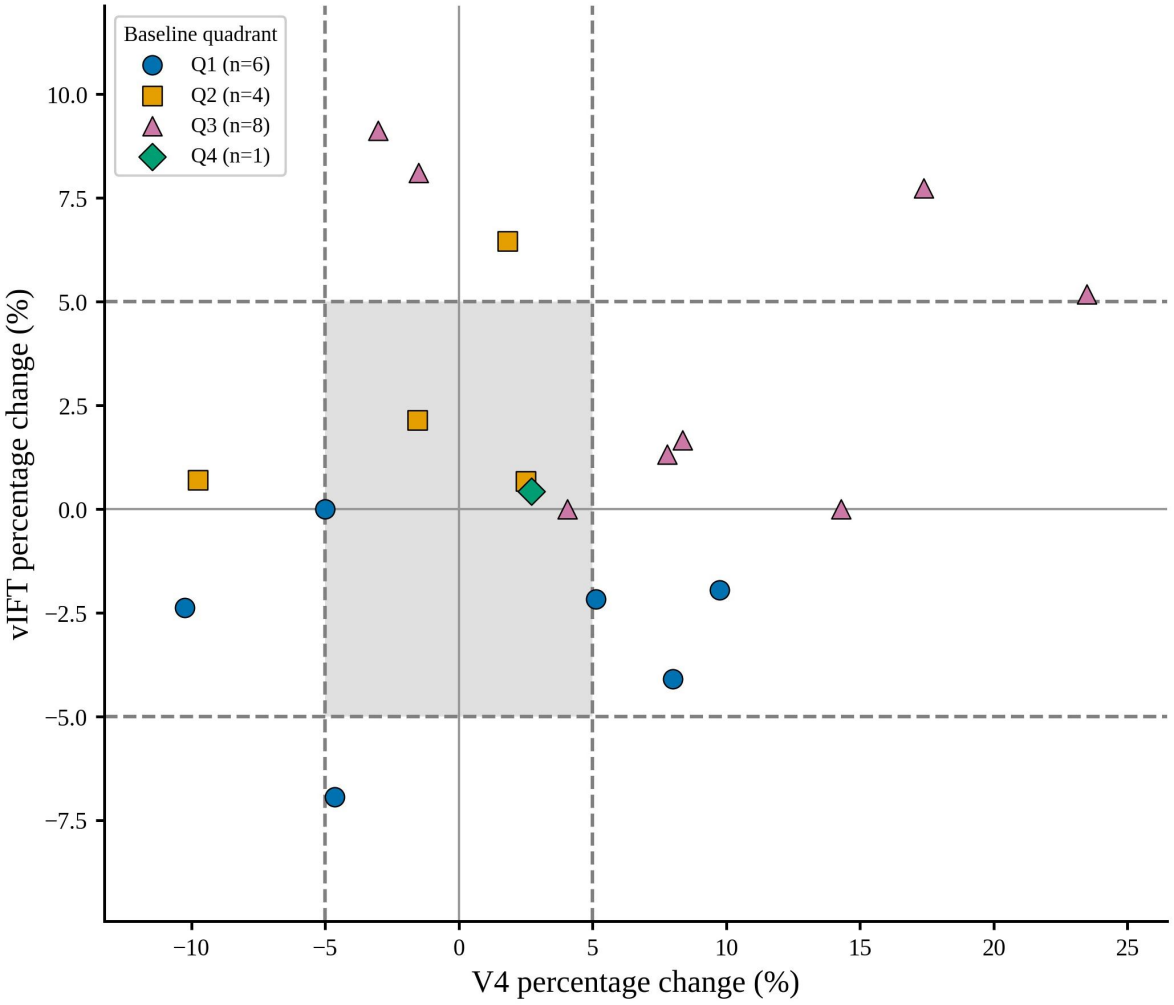
Individual percentage changes in V4 and vIFT relative to CV thresholds in the control group (*n* = 19). Plotting conventions as in Figure 6; quadrant membership is descriptive only (see Figure 3).

**Figure 8:**
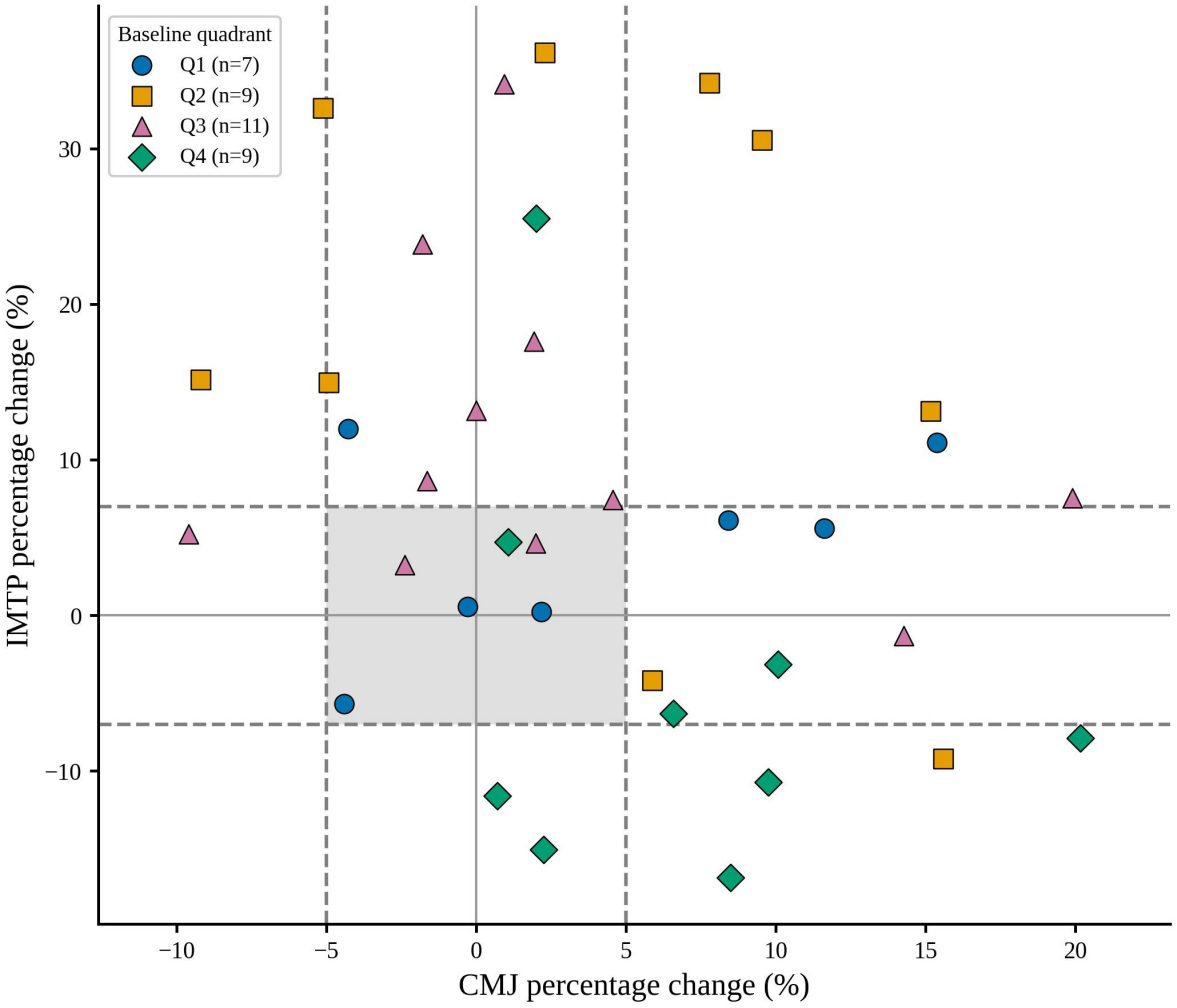
Individual percentage changes in CMJ and IMTP relative to CV thresholds (CMJ: ±5%; IMTP: ±7%) in the intervention group (*n* = 36). Plotting conventions as in Figure 6.

**Figure 9:**
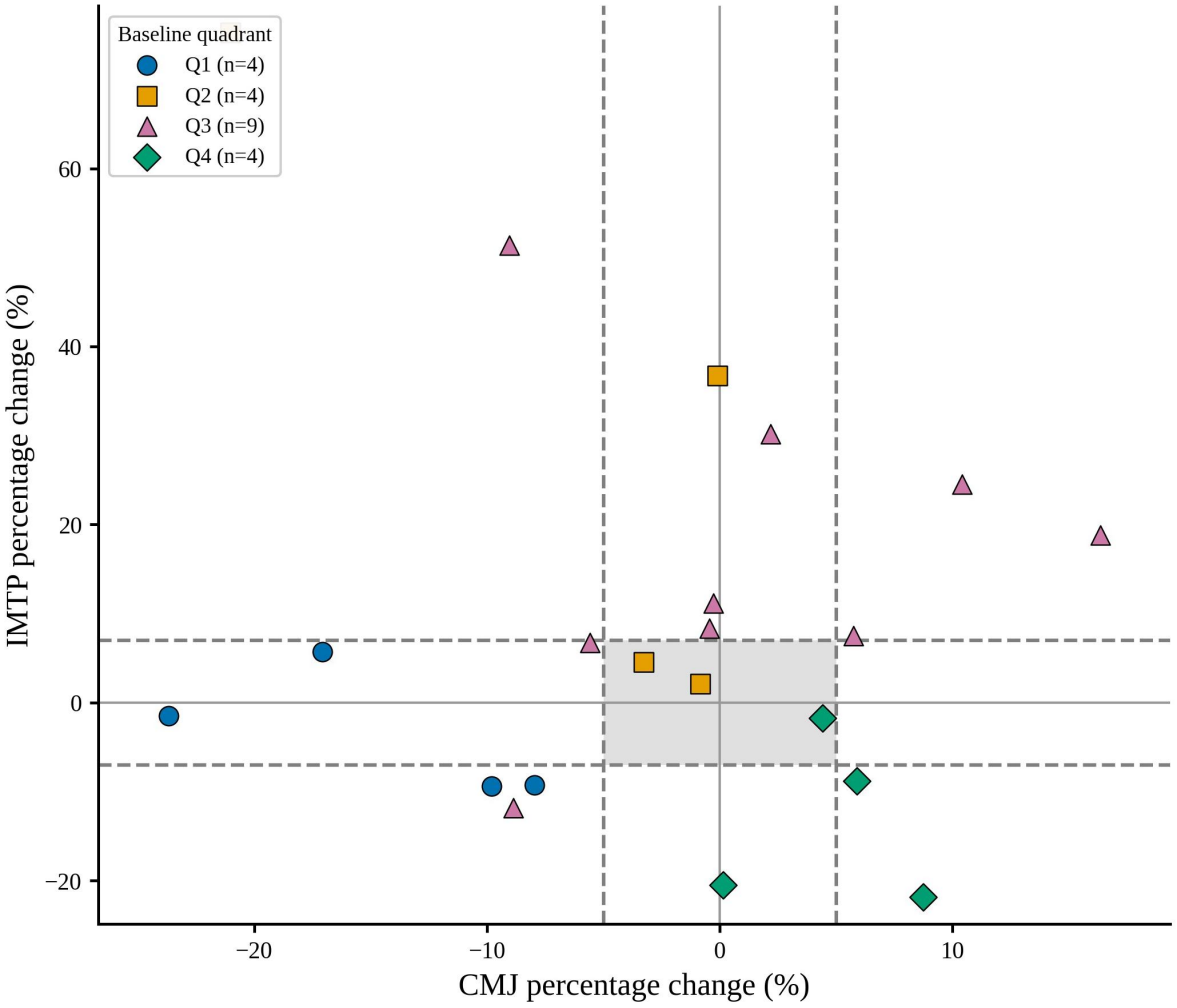
Individual percentage changes in CMJ and IMTP relative to CV thresholds in the control group (*n* = 21). Plotting conventions as in Figure 6; quadrant membership is descriptive only (see Figure 3).

In the intervention group, individual changes exceeding the CV threshold were observed in 55.7% of players for V4, 41.6% for CMJ, and 50.1% for IMTP, whereas 38.6%, 44.9%, and 32.6% of players, respectively, remained within the expected range of measurement variability. Declines exceeding the CV threshold were observed in 5.7% for V4, 13.4% for CMJ, and 17.3% for IMTP. vIFT showed little evidence of individual improvement beyond measurement variability, with only 5.5% of players improving beyond the CV threshold, while 89.5% remained within it and 5.0% declining.

In the control group, CV-based individual responses were more heterogeneous. For V4, 42.0% of players improved beyond the CV threshold, while 34.2% remained unchanged and 23.8% showed a decline. For vIFT, the corresponding proportions were 33.5%, 59.7%, and 6.8%; for CMJ, 19.9%, 45.0%, and 35.1%; and for IMTP, 42.1%, 32.1%, and 25.8%. Consistent with the between-group ANCOVA, the proportion of players exceeding the vIFT improvement threshold was higher in the control group than in the intervention group, providing individual-level corroboration that the observed vIFT trend did not favor the intervention. Consistent with the exploratory, hypothesis-generating nature of these analyses, and given the coefficient-of-variation threshold reflects measurement reliability rather than a criterion for practical or performance relevance, these proportions should not be interpreted as indicating clinically or practically meaningful improvement without further corroboration.

### 3.6 Exploratory quadrant-specific response analysis

Quadrant-specific analyses used the actual baseline quadrant classification applied for individualized training prescription within the intervention group (see Section 2.4), rather than a *post-hoc* statistical reclassification. In the endurance domain, players classified as Q1 (above-average baseline V4 and vIFT, the largest endurance subgroup, *n* = 15) showed a small further V4 increase [+2.44%, 95% CI (−0.80, 5.68)] alongside a decline in vIFT that excluded zero [−2.39%, 95% CI (−4.12, −0.67)], indicating that already high-performing players did not maintain vIFT despite showing continued V4 gains. Players classified as Q3 (low baseline V4 and low baseline vIFT, *n* = 11 for V4, *n* = 10 for vIFT) showed the largest pooled improvement in V4 [+11.49%, 95% CI (1.47, 21.50)], while vIFT increased only modestly [+1.98%, 95% CI (−2.35, 6.31)]. Q4 players (*n* = 6), for whom V4 was the primary target, improved V4 by +9.92% [95% CI (−2.41, 22.25)], whereas vIFT decreased slightly [−1.94%, 95% CI (−8.05, 4.17)]. Q2 players (*n* = 3 for V4, *n* = 2 for vIFT) could not be reliably estimated given the small cell size and are reported descriptively only. CV-based responder classifications broadly supported this pattern, with 87.5% of Q3 players and 78.0% of Q4 players exceeding the V4 threshold.

In the strength domain, Q2 players (*n* = 9), characterized by high CMJ but low IMTP, showed a target-consistent improvement in IMTP [+18.38%, 95% CI (2.80, 33.95); 74.7% exceeding threshold], while CMJ changed modestly [+4.21%, 95% CI (−5.53, 13.95); 56.0% exceeding threshold]. Q3 players (*n* = 11), characterized by low CMJ and low IMTP, also improved IMTP [+11.65%, 95% CI (−2.02, 25.33); 66.2% exceeding threshold], while CMJ changed only modestly [+2.66%, 95% CI (−6.70, 12.03); 23.3% exceeding threshold]. Q4 players (*n* = 9), for whom CMJ was the primary target, showed a positive CMJ change [+6.79%, 95% CI (−4.71, 18.29); 46.7% exceeding threshold], whereas IMTP, which was not the primary target for this quadrant, showed a small decline [−4.60%, 95% CI (−17.56, 8.37); 12.7% exceeding threshold]. Q1 players (*n* = 7), already above average in both variables, showed changes of +4.09% [95% CI (−7.42, 15.59); 45.4% exceeding threshold] for CMJ and +4.27% [95% CI (−12.49, 21.03); 41.4% exceeding threshold] for IMTP. These quadrant-specific analyses are exploratory and hypothesis-generating and should not be interpreted as evidence of statistically established quadrant-specific treatment effects. In particular, given the strong negative correlations identified between baseline performance and subsequent change across all four outcomes (r = −0.36 to −0.64, indicating a substantial regression-to-the-mean signal), the pattern of larger improvements among players with lower baseline values, most evident for V4, cannot be attributed to the quadrant-specific training emphasis with confidence and may partly or wholly reflect regression to the mean rather than a differential training effect. The baseline quadrant allocation, and the corresponding individual pre-to-post changes, are shown in Figures 2, 4, 6, 8. The complete exploratory quadrant-specific response analysis is presented in Table 4.

**Table 4:** Exploratory quadrant-specific percentage changes and CV-based responder rates within the intervention group.

Exploratory Quadrant-Specific Response Analysis						
Baseline Quadrant	Baseline Profile	Primary Training Target	*n*	Outcome	Change, % [95% CI]	CV Responders, *n* (%)
Endurance						
Q1	High V4/High vIFT	maintain/develop V4 and vIFT	15	V4	2.44 [−0.80, 5.68]	3.1 (20.5%)
Q1	High V4/High vIFT	maintain/develop V4 and vIFT	15	vIFT	−2.39 [−4.12, −0.67]	0.1 (0.5%)
Q2	High V4/Low vIFT	target = vIFT	3	V4	5.37 [CI not estimable, *n* < 5]	2.1 (69.3%)
Q2	High V4/Low vIFT	target = vIFT	2	vIFT	0.95 [CI not estimable, *n* < 5]	0.1 (6.0%)
Q3	Low V4/Low vIFT	target = V4 and vIFT	11	V4	11.49 [1.47, 21.50]	9.6 (87.5%)
Q3	Low V4/Low vIFT	target = V4 and vIFT	10	vIFT	1.98 [−2.35, 6.31]	1.7 (17.2%)
Q4	Low V4/High vIFT	target = V4	6	V4	9.92 [−2.41, 22.25]	4.7 (78.0%)
Q4	Low V4/High vIFT	target = V4	6	vIFT	−1.94 [−8.05, 4.17]	0.0 (0.0%)
Strength						
Q1	High CMJ/High IMTP	maintain/develop CMJ and IMTP	7	CMJ	4.09 [−7.42, 15.59]	3.2 (45.4%)
Q1	High CMJ/High IMTP	maintain/develop CMJ and IMTP	7	IMTP	4.27 [−12.49, 21.03]	2.9 (41.4%)
Q2	High CMJ/Low IMTP	target = IMTP	9	CMJ	4.21 [−5.53, 13.95]	5.0 (56.0%)
Q2	High CMJ/Low IMTP	target = IMTP	9	IMTP	18.38 [2.80, 33.95]	6.7 (74.7%)
Q3	Low CMJ/Low IMTP	target = CMJ and IMTP	11	CMJ	2.66 [−6.70, 12.03]	2.6 (23.3%)
Q3	Low CMJ/Low IMTP	target = CMJ and IMTP	11	IMTP	11.65 [−2.02, 25.33]	7.3 (66.2%)
Q4	Low CMJ/High IMTP	target = CMJ	9	CMJ	6.79 [−4.71, 18.29]	4.2 (46.7%)
Q4	Low CMJ/High IMTP	target = CMJ	9	IMTP	−4.60 [−17.56, 8.37]	1.1 (12.7%)

Values are based on pooled percentage changes within baseline quadrants of the intervention group, using the actual quadrant classification applied for training prescription (not a *post-hoc* statistical reclassification). The primary training target indicates the variable or variables directly addressed by the quadrant-specific training emphasis. CV responders refer to players exceeding the predefined coefficient-of-variation threshold for the respective outcome. Quadrant-specific n's reflect players with a valid baseline quadrant classification (requiring both variables in a domain to be observed at baseline) and, for a given outcome, sufficient longitudinal data for that outcome; these n's are therefore a subset of the outcome-specific analytic sample reported in Table 2 (bounded by the smaller of the two domain-specific analytic samples, e.g., *n* = 35 for vIFT in the endurance domain). For Q2 (*n* < 5 for both outcomes), confidence intervals could not be reliably estimated given the small cell size and are reported descriptively only. These analyses are exploratory and hypothesis-generating; given the strong baseline-to-change correlations identified in the quadrant-specific analysis above (Section 3.6), apparent quadrant-specific patterns may partly reflect regression to the mean rather than a differential training effect.

## 4 Discussion

Overall, V4 and CMJ showed small-to-moderate between-group effects in favor of the intervention, whereas the vIFT effect was of a similar magnitude but favored the control group. The between-group effect for IMTP was negligible. However, none of the four outcomes remained statistically significant after correction for multiple comparisons. Among the outcomes, CMJ showed the lowest nominal *p*-value and the most consistent effect across sensitivity analyses, but this finding should be interpreted as preliminary rather than confirmatory given the limited statistical power.

### 4.1 Outcome-specific adaptations to quadrant training

A notable finding of this study was a within-group improvement in V4 in the intervention group that was not observed in the control group, with a between-group effect of moderate size that approached, but did not reach, statistical significance after correction for multiple comparisons (*η*²ₚ = 0.125, adjusted *p* = 0.088). As V4 reflects running velocity at a blood lactate concentration of 4 mmol·L⁻¹, it serves as a practical marker of submaximal endurance performance and metabolic efficiency - qualities particularly relevant in soccer, where players must repeatedly recover between high-intensity actions while sustaining moderate running speeds over prolonged periods (68, 69).

The improvement in V4 suggests a beneficial effect of QT on submaximal endurance capacity. Players with lower V4 values were assigned to MICT, a stimulus known to enhance lactate kinetics, aerobic energy contribution, peripheral oxidative capacity, and running economy (41, 70–72). Together, these adaptations may enable players to sustain higher running speeds at a comparable level of metabolic strain.

Notably, regular soccer training alone did not induce significant V4 improvements in the control group, suggesting that the mixed-intensity nature of soccer practice may provide an endurance stimulus too variable or insufficiently specific to drive threshold-related adaptations (12, 45). QT, by contrast, deliberately matched training mode to each player's identified limiting factor - resulting in a targeted yet practically feasible intervention, although, as noted in Section 4.4, this pattern cannot be distinguished from an unmeasured academy-level effect given the non-randomized design.

These findings support the notion that field-based lactate diagnostics can be translated into profile-specific endurance prescriptions within applied youth soccer settings, where fully individualized programming is often not viable.

The most pronounced strength-related effect was observed for CMJ, an established indicator of explosive lower-body power and stretch-shortening cycle function, i.e., qualities central to jumping, sprinting, acceleration, and rapid change of direction in soccer (73–75). Players classified as below average in CMJ were assigned plyometric and explosive power training, which directly targets rapid force production and stretch-shortening cycle efficiency. CMJ showed the lowest nominal *p*-value and the most consistent effect size across sensitivity analyses of the four outcomes (*η*²ₚ = 0.136), which is suggestive that this training allocation may have contributed to the observed pattern, although this effect did not survive correction for multiple comparisons (adjusted *p* = 0.078) and should therefore be interpreted with caution. This interpretation is further complicated by a significant baseline-by-group interaction: the apparent between-group difference in CMJ was negligible among players with lower baseline values and increased substantially among players with higher baseline CMJ, a pattern that does not straightforwardly support the notion that quadrant-targeted training preferentially benefited players with the lowest initial CMJ.

The absence of comparable improvements in the control group suggests that regular soccer training alone may not provide a sufficient or specific enough overload to enhance explosive power during the competitive season. Although soccer practice inherently includes jumps, sprints, and accelerations, these actions are unlikely to be performed with the volume, progression, or neuromuscular specificity required to elicit measurable CMJ improvements (6, 10, 76). Similar improvements in CMJ following plyometric or neuromuscular training interventions have also been reported in several other studies (76–78), consistent with these findings across different cohorts and training contexts.

In contrast to V4, vIFT was less responsive to QT in this cohort and setting. Although players with lower vIFT were assigned to HIIT, the applied dose may have been insufficient to drive further adaptation in already trained youth players, as meaningful improvements in maximal intermittent fitness likely require adequate intensity, volume, progression, and recovery (79, 80). Regular soccer training already incorporates frequent high-intensity bouts through small-sided games and match play, potentially diminishing the marginal adaptive stimulus of supplementary HIIT and complicating the isolation of intervention effects from routine training demands (42, 81–83).

The concurrent training load during the competitive season may have further contributed to this outcome. Simultaneous exposure to soccer practice, match play, strength training, and supplementary endurance sessions may have limited adaptive capacity or attenuated maximal performance expression during testing (84, 85). This is particularly important for vIFT, which demands repeated high-intensity efforts and volitional exhaustion, rendering test performance susceptible to fatigue accumulation and motivational variability (86, 87) - a susceptibility to verbal encouragement and assessor-related motivational bias that, in principle, applies equally to the maximal-effort CMJ and IMTP assessments, given the unblinded testing noted in Section 4.4.

A further methodological consideration concerns the stage-based structure of the 30-15IFT, which may limit sensitivity to small performance changes. Genuine physiological improvements smaller than one full stage increment may remain undetected, as increases in vIFT are only registered when a player completes an additional 30-s stage and advances to the next velocity level, i.e., adaptations below the test's resolution (e.g., <1 km·h⁻¹) may not be captured in the recorded outcome, an insensitivity that was also evident in the CV-based individual response analysis (66, 88).

IMTP reflects maximal strength relevant to sprint acceleration, physical duels, change of directions and overall force capacity (33, 89). Evidence for an intervention effect on maximal strength was, however, less conclusive: although the intervention group demonstrated a significant within-group improvement in relative IMTP, the absence of a significant between-group effect limits its interpretation as a clear intervention-induced adaptation. One additional heavy resistance training session per week may have been insufficient to elicit greater improvements in maximal strength, which typically requires progressive overload, repeated training exposure, and adequate recovery. Notably, these conditions are difficult to fulfill during the competitive season when strength training is performed concurrently with soccer practice, match play, and supplementary endurance training (90, 91).

### 4.2 Individual responses beyond measurement variability

The CV-based individual response analysis indicates that a majority of players in the intervention group exceeded the CV threshold for improvement in V4 (55.7%), IMTP (50.1%), and CMJ (41.6%), whereas individual vIFT changes were largely within normal measurement variability (5.5% improved). Notably, a larger proportion of players in the control group exceeded the vIFT improvement threshold (33.5%) than in the intervention group, corroborating the between-group ANCOVA finding that the observed vIFT trend did not favor the intervention. For the other outcomes, the control group showed a more heterogeneous individual response profile, with improvements in some players accompanied by substantial proportions of unchanged or declined responses.

These findings point to the potential practical value of QT as a group-based individualization strategy, particularly where the prescribed stimulus was closely aligned with the targeted limitation, while acknowledging that CV-based responder classification reflects change exceeding measurement variability and does not by itself establish practical or performance relevance. The V4 response pattern suggests that directing players with lower submaximal endurance profiles toward targeted endurance work was associated with changes exceeding normal test-retest variability in a majority of players. Individual-level changes exceeding measurement variability in CMJ and IMTP similarly suggest that quadrant-specific neuromuscular training may be associated with adaptations in explosive power and maximal strength, although, as noted above, this CV-based classification does not by itself establish practical or performance relevance. The weaker vIFT response likely reflects both the high-intensity demands already embedded in regular soccer training and the limited resolution of the stage-based 30-15IFT scoring structure. Collectively, the CV-based analysis contextualizes the group-level findings by demonstrating that individual-level change was not uniformly distributed within groups, and that even outcomes without a statistically confirmed between-group effect (e.g., IMTP) included a substantial proportion of individual responders.

### 4.3 Quadrant-specific response patterns

The exploratory quadrant-specific analysis extends the group-level findings by examining whether adaptations aligned with the intended training emphasis of each baseline profile. The most pronounced target-aligned pattern was observed for V4, particularly in endurance quadrants characterized by low baseline V4. However, this pattern must be interpreted with substantial caution: strong negative correlations between baseline performance and subsequent change were identified across all four outcomes (r = −0.36–−0.64), indicating a considerable regression-to-the-mean signal that plausibly accounts for part or all of the apparent target-aligned improvement among players with the lowest baseline values, independent of any differential training effect. By contrast, vIFT showed limited evidence of a profile-specific response, suggesting that the quadrant-based endurance prescription was more effective for improving submaximal aerobic performance than maximal intermittent fitness, although this comparison is subject to the same regression-to-the-mean caveat. In the strength domain, target-aligned adaptations were observed for IMTP in Q2 and Q3, whereas CMJ improvements were not restricted to players with low initial CMJ and instead appeared more pronounced among players with higher baseline CMJ, consistent with the significant baseline-by-group interaction identified in the primary ANCOVA. Taken together, these exploratory findings should not be interpreted as evidence of statistically established quadrant-specific treatment effects. They are hypothesis-generating and were based on small and unequal subgroup sizes (including cells with fewer than five players, for which confidence intervals could not be reliably estimated), and the observed patterns, particularly for V4, may partly or wholly reflect regression to the mean rather than a genuine differential training effect.

### 4.4 Strengths, limitations and future directions

The core strength of QT lies in its practical simplicity. Classifying players along two variables allows coaches to identify performance-limiting factors and assign targeted training without individual plans for every player, which represent a meaningful advantage in youth academies with limited staff, technology, and time (56, 92). This transparency may also improve interdisciplinary communication and player buy-in (93), as the visualization of performance-limiting factors within a simple and intuitive framework facilitates understanding and decision-making among coaching staff and players alike (92, 94) as well as improves the acceptance of training interventions, as players can better understand the rationale behind their assigned training emphasis (95). The close alignment between the prescribed training stimulus and each player's targeted outcome may also help explain why the most consistent effects were observed for V4 and CMJ, although this potential mechanism was not directly tested in the present study. As a potential future application requiring separate validation, profiling players against team-level norms might inform role allocation, tactical adjustments, and recruitment decisions where individual physical profiles diverge from the demands of a given playing style (96, 97). Repeated testing further enables dynamic adjustment of quadrant allocation across the season.

Several limitations warrant consideration, most importantly that intervention allocation occurred at academy level, with only one academy per condition and no randomization; academy and intervention condition were therefore fully confounded, and the design cannot distinguish QT effects from academy-specific influences such as coaching practices, culture, or facilities. Unequal, incomplete samples and uncontrolled confounders, including maturation status, biological age, training history, and isometric testing familiarity, further limit the strength of conclusions (98–101). The control group was also descriptively older and taller than the intervention group at baseline (Table 1); given the modest sample size relative to the number of primary outcomes, age and height were not entered as additional ANCOVA covariates beyond each outcome's own baseline value, so residual confounding from this imbalance cannot be excluded. The control group's continued participation in general strength exercises further reduced between-group contrast, while accumulated training and match demands during the competitive season may have attenuated adaptations or impaired performance expression during testing (102–105). Although football-session frequency and duration were matched, total training exposure was not, so differences in volume or additional load may have confounded the intervention effect. Profiling players against internal team norms may introduce team-specific bias and obscure differences relative to broader competitive, positional, or age-group standards, though this also has a practical advantage, as players were classified under identical conditions (106–110). Reducing player profiles to two variables risks oversimplifying a multidimensional performance construct, and dichotomous classifications may fail to capture the context-dependent expression of physical qualities in soccer performance (97, 111). Position-specific clustering could offer a more differentiated alternative, as direct comparisons between positions may be misleading given their substantially different physical profiles (7, 96). Stage-based vIFT scoring may not capture small physiological adaptations, and misclassification from measure insensitivity remains a concern; randomized designs, larger samples, longer interventions, and stricter load monitoring would help future studies distinguish insufficient dose, fatigue, test insensitivity, and true non-response. Statistical power was limited: none of the four primary between-group effects survived correction for multiple comparisons, and the exploratory quadrant-specific analyses were particularly underpowered, with some subgroups below five players. Target-aligned improvements among lower-baseline players, especially for V4, coincided with strong negative baseline-to-change correlations across all outcomes, so regression to the mean cannot be excluded; findings for V4, vIFT, and CMJ are best characterized as suggestive of genuine, if imprecisely estimated, effects rather than statistically confirmed outcomes. The study was also not prospectively registered: the four primary outcomes were specified before the analytic dataset was examined, but not via a public registry; we report this transparently rather than presenting it as pre-registration. Outcome assessors were not blinded to group allocation, as testing was partly conducted by academy-affiliated staff, a further potential source of bias. An *a priori* sample-size and power calculation was performed; the achieved sample was smaller than the pre-specified target due to academy or team availability changes and missing assessments from illness, injury, absence, or inability to complete specific tests. Adverse events were not independently monitored but were managed internally by academy coaching staff, with no intervention-related injuries or dropouts reported to us. Playing position was not systematically recorded and could not be examined as a potential influence on baseline profiles or quadrant allocation, a further unmeasured source of variability. Adherence to the prescribed QT sessions was systematically overseen by academy staff but not logged, so formal attendance or compliance figures cannot be reported; undocumented adherence variation could have contributed to individual differences in response. Attendance and training-load data for regular soccer sessions were similarly not recorded for either group. Finally, because quadrant classification is defined relative to the team mean of the sample tested, thresholds, and therefore quadrant membership, are inherently sample-dependent: a player classified as “high” in one team could be classified as “low” in another with the same absolute performance. This limits the transferability and stability of quadrant classification across teams, age groups, or competitive levels and should be considered when applying the QT framework beyond the present sample.

### 4.5 Practical applications

Effect sizes for V4 and CMJ were consistent with a small-to-moderate beneficial training effect on submaximal endurance capacity and explosive lower-body power. CMJ showed the most precise pattern across sensitivity analyses, though this effect was itself baseline-dependent, negligible at low baseline values and more pronounced at higher ones, and, like the other outcomes, did not survive correction for multiple comparisons; all findings should therefore be regarded as preliminary. As such, CMJ should not be read as a uniformly favorable finding but as one whose apparent benefit was concentrated among players with higher baseline values. The vIFT effect was of similar magnitude but favored the control condition, while the effect on IMTP was negligible. The limited and statistically unconfirmed nature of these findings likely reflects a combination of modest available statistical power, concurrent training demands, accumulated fatigue, and, for the quadrant-specific patterns in particular, a plausible contribution from regression to the mean. The approach seems to be most effective when training stimuli are sufficiently specific, progressive, and closely aligned with the targeted performance outcome. CV-based individual response classification can further help coaches identify which players show meaningful adaptation and which may require an adjusted emphasis.

In practice, QT may serve as a practical decision-making framework, particularly in settings with limited staffing and resources, though its value as a means of producing statistically confirmed performance improvements remains to be established in adequately powered, randomized trials. Rather than being applied as a fixed label, it should function as a flexible monitoring tool that is updated after repeated testing to adjust training emphasis across the season. Future studies should incorporate longer and more outcome-specific interventions, larger and position-stratified samples, maturation status, and more standardized testing conditions to improve measurement sensitivity and clarify the boundaries of this approach.

## Data Availability

The datasets presented in this article are not readily available because of privacy and ethical restrictions involving youth athletes but are available from the corresponding author on reasonable request. Requests to access the datasets should be directed to timo.steidle@stud-mail.uni-wuerzburg.de.
