# Supplementary material for: Quadrant training: a simple typology-based approach to improve strength and endurance in youth soccer players
Source: Front Sports Act Living. 2026 Oct 7;8:1936767. doi: 10.3389/fspor.2026.1936767 (PMC13683625; doi:10.3389/fspor.2026.1936767)
Supplement: Supplementary file 1 [file Table1.pdf]

Supplementary Material

Supplementary Table 1. Comparison of naive vs. robust standard errors for the primary between-group ANCOVA model, a multiple-imputation sensitivity analysis restricted to players with data at all three testing time points, and complete-case (non-imputed) within-group comparisons.

Supplementary Table 1a. Between-group effect estimates for the primary multiple-imputation model, comparing naive (model-based) and robust (HC3) standard errors.

Naive vs. Robust (HC3) Standard Errors						
**Outcome**	**b**	**Naive SE**	**Naive p**	**Robust (HC3) SE**	**Robust p**	**adj. p (BH), robust**
**V4**	−0.201	0.097	0.047	0.105	0.066	0.088
**vIFT**	0.620	0.289	0.045	0.304	0.054	0.088
**CMJ**	−2.778	1.127	0.018	1.139	0.020	0.078
**IMTP**	−0.680	0.921	0.466	0.975	0.490	0.490

Note. b = unstandardized between-group effect estimate from the ANCOVA model (POST outcome regressed on PRE outcome and group, intervention vs. control), pooled across imputed datasets using Rubin's rules. Naive SE = model-based pooled standard error; Robust (HC3) SE = heteroscedasticity-consistent standard error, used as the primary inference criterion in this study (see Section 2.6 and Table 4). adj. p (BH) = Benjamini-Hochberg-corrected p-value across the four primary outcomes, robust SE only. Naive and robust point estimates (b) are identical because robust SE affects only the standard error and p-value, not the estimate itself.

Supplementary Table 1b. Sensitivity analysis: between-group effect estimates from a multiple-imputation model restricted to players with observed data at all three testing time points (PRE, MID, POST).

Sensitivity Analysis (All Three Time Points)				
**Outcome**	**b**	**SE**	**95% CI**	**p**
**V4**	−0.183	0.090	[−0.369, 0.002]	0.052
**vIFT**	0.371	0.205	[−0.049, 0.791]	0.081
**CMJ**	−2.474	1.009	[−4.519, −0.429]	0.019
**IMTP**	−0.776	0.903	[−2.616, 1.064]	0.396

Note. Between-group ANCOVA estimates (POST on PRE and group) from a sensitivity multiple-imputation model restricted to players with observed data at all three testing time points (PRE, MID, POST), excluding players missing at all three occasions for the respective outcome. Naive (model-based) standard errors are shown; this sensitivity model was not re-estimated with robust (HC3) standard errors. Estimates are directionally consistent with the primary model (Table 4).

Supplementary Table 1c. Complete-case (non-imputed) within-group paired pre-post comparisons, by group.

Complete-Case Paired Pre-Post Comparisons					
**Outcome**	**Group**	**n**	**Δ**	**95% CI**	**p**
**V4**	Intervention Group	18	+0.183	[0.067, 0.299]	0.004
**V4**	Control Group	10	+0.000	[−0.224, 0.224]	1.000
**vIFT**	Intervention Group	18	−0.250	[−0.509, 0.009]	0.058
**vIFT**	Control Group	6	+0.083	[−0.689, 0.856]	0.793
**CMJ**	Intervention Group	20	+1.327	[0.151, 2.503]	0.029
**CMJ**	Control Group	9	+0.260	[−0.952, 1.472]	0.634
**IMTP**	Intervention Group	20	+1.884	[0.806, 2.962]	0.002
**IMTP**	Control Group	8	+0.919	[−1.013, 2.850]	0.298

Note. Paired-samples t-tests on observed (non-imputed) pre- and post-intervention values, complete cases only (players with both PRE and POST measurements for the respective outcome). These within-group comparisons are descriptive and do not adjust for baseline differences between groups; the primary between-group inference is reported in Table 4. Consistent with the multiply imputed analyses, the intervention group showed significant improvements in V4, CMJ, and IMTP but not vIFT, while the control group showed no significant pre-post changes for any outcome.
